# Blood monocyte and dendritic cell profiles among people living with HIV with *Mycobacterium tuberculosis* co-infection

**DOI:** 10.1186/s12865-023-00558-z

**Published:** 2023-07-21

**Authors:** Santhuri Rambaran, Thando Glory Maseko, Lara Lewis, Razia Hassan-Moosa, Derseree Archary, Sinaye Ngcapu, Nigel Garrett, Lyle R. McKinnon, Nesri Padayatchi, Kogieleum Naidoo, Aida Sivro

**Affiliations:** 1 Centre for the AIDS Programme of Research in South Africa (CAPRISA), Durban, South Africa; 2 South African Medical Research Council (SAMRC)-CAPRISA-TB-HIV Pathogenesis and Treatment Research Unit, University of KwaZulu-Natal Nelson R Mandela School of Medicine, Durban, South Africa; 3 Centre for Tuberculosis, National Institute for Communicable Diseases, Johannesburg, South Africa; 4 Department of Medical Microbiology, University of KwaZulu-Natal, Durban, South Africa; 5 School of Nursing and Public Health, Discipline of Public Health Medicine, University of KwaZulu-Natal, Durban, South Africa; 6 Department of Medical Microbiology and Infectious Diseases, University of Manitoba, Winnipeg, Canada; 7 Department of Medical Microbiology, University of Nairobi, Nairobi, Kenya; 8 JC Wilt Infectious Disease Research Centre, National Microbiology Laboratory, Public Health Agency of Canada, Winnipeg, MB Canada

**Keywords:** Pulmonary-tuberculosis, Inflammation, HIV, Monocytes, Dendritic cells

## Abstract

**Background:**

Understanding the complex interactions of the immune response mediated by *Mycobacterium tuberculosis* and HIV co-infection is fundamental to disease biomarker discovery, vaccine, and drug development. Using flow cytometry, we characterized the frequencies and phenotypic differences in monocytes and dendritic cell populations using peripheral blood mononuclear cells from individuals with recurrent, active pulmonary tuberculosis with and without coexisting HIV infection (CAPRISA 011, Clinicaltrials.gov, NCT02114684, 29/01/2014) and compared them to samples from HIV positive individuals and healthy controls. Additionally, we assessed the associations between the frequency of monocyte and dendritic cell subsets and time to culture conversion and cavitary disease in patients with active TB using a cox proportional hazards and logistic regression models.

**Results:**

Compared to healthy controls, the frequency of total monocytes (HLA-DR + CD14 +) was significantly higher in the TB/HIV and TB groups and the frequency of dendritic cells (HLA-DR + CD14-) was significantly higher in TB/HIV and HIV groups. We observed significant variation in the expression of CCR2, CD40, CD11b, CD86, CD163, CX3CR1 across different cell subsets in the four study groups. Increase in CCR2, CD11b and CD40 was associated with active TB infection, while decrease in CX3CR1 and increase in CD163 was associated with HIV infection. Expression of CX3CR1 (aHR 0.98, 95% CI 0.963 – 0.997, *p* = 0.019) on non-classical monocytes associated with longer time to TB culture conversion in the multivariable model correcting for randomization arm, age, sex, HIV status, lung cavitation, alcohol use, smoking and BMI. Higher surface expression of CD86 (aOR 1.017, 95% CI 1.001 – 1.032, *p* = 0.033) on intermediate monocytes associated with the presence of lung cavitation, while higher expression of transitional monocytes (aOR 0.944, 95% CI 0.892 – 0.999, *p* = 0.047) associated with the absence of lung cavitation in the multivariable model.

**Conclusion:**

These data provide valuable insight into the heterogenous role of monocyte and dendritic cells in TB and HIV infections.

**Supplementary Information:**

The online version contains supplementary material available at 10.1186/s12865-023-00558-z.

## Introduction

Despite being a treatable and preventable disease, tuberculosis (TB) continues to claim millions of lives each year and remains the leading cause of mortality among HIV positive individuals, causing approximately a third of HIV-1 associated deaths. At the same time HIV infection is the strongest risk factor for TB infection progressing to TB disease, with people living with HIV (PLWH) being approximately 18 times more likely to develop TB disease compared to people without HIV [1]. There continues to be an urgent need for novel approaches for diagnosis, treatment and prevention of TB in PLWH, especially in sub-Saharan Africa, where the syndemic interaction between TB and HIV is felt most acutely. Identification of immunological correlates of protection and risk for TB can aid in evaluation and development of novel vaccine candidates, new diagnostic methods and the development of shorter treatment regiments [2]. While significant work has been done in characterizing host immune response in TB and HIV infections individually, the innate and adaptive immune responses in TB/HIV co-infection remain poorly understood. HIV mediated chronic inflammation and the impairments in the innate and adaptive immunity are known to contribute to increased risk of TB in PLWH [3]. In our previous studies we have shown that increase in systemic inflammatory markers are a strong predictor of TB reactivation in HIV positive individuals [4, 5]. Immune activation and dysregulation of monocyte/macrophages and dendritic cell phenotype and function is known to contribute to both HIV and TB pathogenesis [6–9]. During TB and HIV infection, monocytes, and dendritic cells (DCs) can be activated due to increased bacterial/viral load, microbial translocation and gastrointestinal damage, and increased production of pro-inflammatory cytokines. Changes in their phenotype can affect cell migration and tissue distribution, activation of the adaptive immune response and lead to further increase in the inflammatory response, all of which can impact pathogen clearance and immunopathogenesis [10]. Phenotypic and functional alterations in circulating monocytes and dendritic cells have been reported from patients with pulmonary TB [11] and these changes are thought to play a role in bacterial persistence [12–14]. Enhanced monocyte activation was observed in PLWH with latent TB infection or prior active TB and could contribute to the pathogenesis of non-communicable disease in HIV [15].

A better understanding of the intricate and dynamic interactions between the host immune response and *Mycobacterium tuberculosis* (Mtb) in the context of HIV co-infection is crucial to the development of better drugs and vaccines for the prevention and treatment of TB in HIV co-infected individuals. In this study, we set out to characterize the phenotypic differences in monocyte and DC subsets during active pulmonary TB in HIV-positive and HIV-negative individuals from South Africa.

## Materials and methods

### Study population

Stored peripheral blood mononuclear cells (PBMCs) used for this study were from the CAPRISA 011 Improving Retreatment Success (IMPRESS) trial. This was an open label, randomized clinical trial to determine if treatment outcomes would be improved with the substitution of moxifloxacin [HRZM (isoniazid, rifampicin, pyrazinamide, and moxifloxacin), active arm] for ethambutol [HRZE (isoniazid, rifampicin, pyrazinamide and ethambutol), control arm] in patients with recurrent TB. CAPRISA 011 study participants were recruited and treated at an urban clinic operated by the Centre for the AIDS Programme of Research in South Africa (CAPRISA) eThekwini Research Clinic that adjoins the largest government outpatient HIV-TB facility, the Prince Cyril Zulu Communicable Disease Centre (PCZCDC) in KwaZulu-Natal (KZN), South Africa (SA) [16]. Participants enrolled in the study were adults ≥ 18 years who had a previous history of TB treatment completion, with rifampicin-susceptible Mtb and were sputum smear-positive by GeneXpert MTB/RIF® technology. Patients were monitored with 2-weekly clinical follow-up visits during the 8 weeks of intensive phase of TB treatment, and a monthly clinical follow-up during the 16 weeks of continuous phase of TB treatment. Patient enrolment started in November 2013 and follow-up ended in July 2017.

We conducted a cohort analysis that included 90 individuals with available PBMC samples taken during active TB (baseline), 60 were HIV positive and 30 HIV negative. As controls, we also analysed PBMCs from 19 HIV positive individuals without TB from the CAPRISA 002 Acute HIV Infection study, an observational cohort study aiming to identify viral immune and host genetic factors that predict disease progression [17], and from PBMC samples from 11 healthy donors.

### Sample collection and processing

Peripheral blood was collected in acid citrate dextrose (ACD) tubes. PBMCs were isolated by Ficoll-Hypaque density gradient centrifugation and cryopreserved in fetal bovine serum (FBS) containing 10% dimethyl sulfoxide (DMSO) in liquid nitrogen for long-term storage.

### Flow cytometry experiments

Thawed PBMCs were washed and resuspended in 5 ml R10 (RPMI 1640 supplemented with 10% FBS, 100 U/ml penicillin, 100 µg/ml streptomycin 1.7 nM sodium glutamate and 5.5 mL HEPES buffer). Cells were rested for 3 h at 37 °C, 5% CO_2_ before staining.

A total of 1 × 10^6^ cells were surfaced stained in the dark at room temperature for 20 min with a panel of conjugated antibodies: anti-CD3 Alexa Fluor 700, anti-CD56 Alexa Fluor 700, anti-CD19 Alexa Fluor 700, anti-CD11c PE-Cy5, anti-HLA-DR PE CF594, anti-CD14 APC Cy7, anti-CD123 BV 421, anti-CD16 BV 785, anti-CD86 BV 650, anti-CD40 BV 711, anti-CD163 PE, anti-CD11b PE-Cy7, anti-CX3CR1 PerCP Cy5.5, anti-CCR2 Alexa Fluor 647 and Live/Dead™ fixable aqua dead cell stain (Sup. Table 1). Following staining, cells were washed twice with PBS-1 and fixed with 1 X CellFix™ (BD, 340,181). Cells were acquired on the BD Fortessa flow cytometer with BD FACSDiva software (v8.0.2). At least 200 000 events were collected. Flow cytometry data was analysed by hierarchal gating using FlowJo software and exported to Excel.

### Flow cytometry gating

Representative gating is shown in Sup. Fig. 1. Fluorescence minus one (FMO) control tubes were used to define gates for select markers (Sup. Fig. 2). Total monocytes expression was classified as HLA-DR^+^CD14^+^. Based on the phenotypic dichotomous expression of surface markers CD14 and CD16 human monocyte subpopulations were classified into three major subsets: classical (CD14^++^CD16^−^, CM), intermediate (CD14^++^CD16^+^, IM] and non-classical (CD14^+^CD16^++^, NCM) [18]. A fourth monocyte subset was observed, described as transitional monocytes (CD14^+^CD16^−^, TM) [19, 20].

Total dendritic cells expression was classified as HLA-DR^+^CD14^−^. Circulating DCs were differentiated into cells of 2 lineages: myeloid (CD123^−^CD11c^+^, mDC) or plasmacytoid (CD123^+^CD11c^−^, pDC) [21]. Additionally, we observed and gated on an additional subset: CD123^dim^CD11c^++^ previously described as early percussors of mDCs [22, 23].

### Statistical analysis

Statistical analyses were performed using IBM SPSS Statistics version 27, SAS version 9.4 and graphs were made using GraphPad Prism (V9.3.1).

D’Agostino-Pearson omnibus normality test was used to assess data distribution. To assess differences in frequencies of monocytes and dendritic cells among TB/HIV, TB, HIV and healthy donors, a one-way ANOVA with Tukey’s post-test was performed on normally distributed markers, and non-parametric Kruskal Wallis test with Dunn’s post-test was done on markers which were not normally distributed.

In the IMPRESS trial, a Cox proportional hazards model was used to determine the association between measured cell population frequencies at baseline and time to culture conversion (first of two consecutive negative TB culture results), measured in days. To determine the association between measured cell population frequencies at baseline with cavitary disease, a logistic regression model was used with presence of lung cavitation at baseline as the outcome. Multivariable analyses adjusted for a wide range of baseline clinical and demographic variables including randomization arm, age, sex, HIV status, lung cavitation, alcohol use, smoking and BMI. In addition, when analysing HIV positive individuals CD4 T cell counts, viral load and ART duration were adjusted for. Randomization arm was excluded in the multivariable lung cavitation analysis as this was not relevant for the studied timepoint. The *p*-values from cox proportional hazards and logistic regression model are reported and discussed as is [24]. The q-value resulting from the original FDR method of Benjamini-Hochberg (FDR, Q = 5%) is included in the supplemental tables.

## Results

### Cohort characteristics

Samples from 90 CAPRISA 011 (IMPRESS) study participants were included in the analysis (Table 1). The median age of the study participants was 34.5 [interquartile range (IQR) 29.0 – 41.5] and 76% were male. The median body mass index (BMI) was 19.4 (IQR 18.2 – 21.7). Of the 90 participants, 60 were HIV positive with a median CD4 cell count of 248 cells/mm^3^ (IQR 150 – 446).

**Table 1:** Demographic and clinical characteristics of the study participants

Variables	IMPRESS Total participants *n* = 90	IMPRESS TB/HIV positive *n* = 60	IMPRESS TB positive and HIV negative *n* = 30	CAPRISA 002 HIV positive *n* = 19	Healthy Donors *n* = 11
**Randomization arm n (%)**					
HRZE—Control	43 (48.0)	28 (47.0)	15 (50.0)	-	-
HRZM—Active	47 (52.0)	32 (53.0)	15 (50.0)	-	-
**Age (y), median (IQR)**	34.5 (29.0 – 41.5)	35.0 (31 – 40.7)	33.0 (24.0 – 50.5)	33.0 (29.0 – 36.0)	34.0 (32.0 – 40.0)
**Gender, n (%)**					
Male	68 (76.0)	42 (70.0)	26 (87.0)	-	5 (45.0)
Female	22 (24.0)	18 (30.0)	4 (13.0)	19 (100.0)	6 (55.0)
**Body mass index (kg/m**^**2**^**), mean (IQR)**	20.6 (18.2 – 21.8)	20.5 (17.9 – 22.3)	20.9 (18.4 – 21.5)	32.9 (30.5 – 35.9)	24.7 (19.7 – 31.3)^a^
**CD4 cell count (cells/mm**^**3**^**), mean (IQR)**	303.0 (150.0 – 446.0)	303.0 (150.0 – 446.0)	-	551.0 (361.0 – 717.0)	-
**HIV viral load (copies/ml), mean (IQR)**	94,907.0 (20.0 – 60,744.0)	94,907.0 (20.0 – 60,744.0)	-	56,399.0 (998.0 – 95,212.0)^c^	-
**ART status n (%)**					
Yes	33 (50.0)	30 (50.0)	-	11 (58.0)	-
No	27 (45.0)	27 (45.0)	-	8 (42.0)	-
**Time on ART (months), median (IQR)**	1.0 (0.0 – 36.0)	1.0 (0.0 – 36.0)	-	5 .0(0.0 – 46.0)	-
**Lung Cavities n (%)**					
None	25 (28.0)	21 (35.0)	4 (13.0)	-	-
One Lung	38 (42.0)	25 (42.0)	13 (43.0)	-	-
Both Lungs	27 (30.0)	14 (23.0)	13 (43.0)	-	-
**Days to first negative solid culture, median (IQR)**^**b**^	54.0 (28.0 – 79.8)	44.0 (28.0 – 79.8)	56.0 (42.0 – 82.3)	-	-
**Alcohol Use in the past 3 months n (%)**					
Yes	29 (32.0)	22 (37.0)	7 (23.0)	-	-
**Smoking in past 3 months n (%)**					
Yes	35 (39.0)	21 (35.0)	14 (47.0)	-	-

^a^1 participant missing BMI

^b^Measures for all variables, except days to first negative culture are reported at baseline

^c^7 missing viral load

The median age of HIV positive participants from the CAPRISA 002 cohort was 33 (29 – 36). All participants were female with a median BMI of 32.7 (IQR 30.4 – 35.9) and a median CD4 cell count of 583 (IQR 361 – 717). Median age of healthy donors was 34 (32 – 40) and 45% were male.

### TB and HIV related differences in monocyte and dendritic cell frequencies and phenotypes

**Fig. 1:**
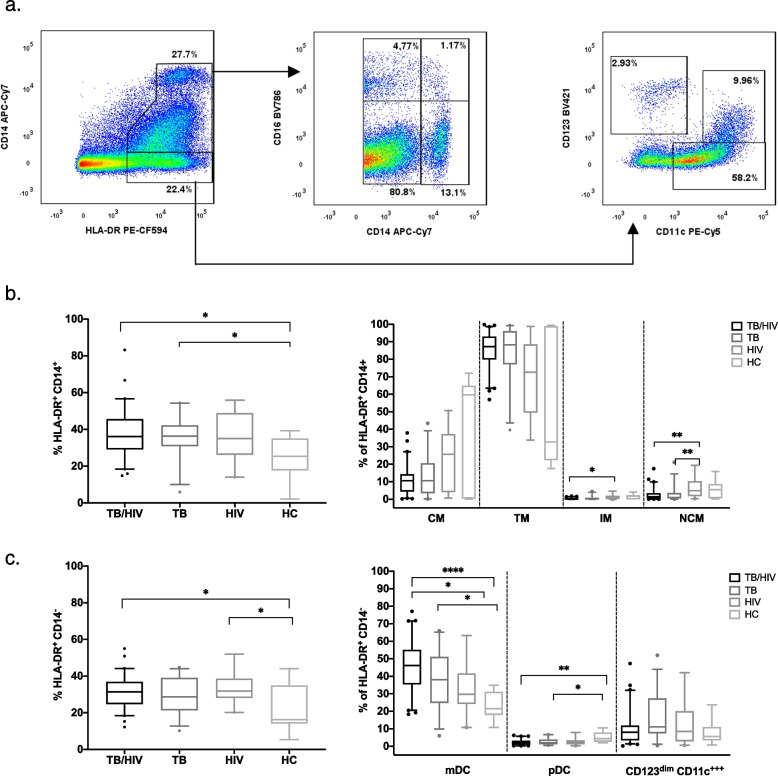
**a** Representative gating of HLA-DR^+^ CD14^+^ (monocyte) and HLA-DR^+^ CD14^−^ (dendritic) cell populations. The staining profile of PBMCs from a representative TB/HIV co-infected participant is shown. **b** Differences in frequencies of total monocytes and monocyte subsets (classical, non-classical, intermediate, and transitional) between study groups (TB/HIV, TB, HIV and HC). **c** Differences in frequencies of dendritic cells and their subsets (mDC, pDC, and CD123^dim^ CD11c.^++^) between study groups. **b** and **c** Boxes represent median and interquartile ranges; whiskers represent 5–95 percentiles. Differences in cell population frequencies among the groups were assessed using a Kruskal–Wallis test with Dunn’s post-test for data that were not normally distributed and one-way ANOVA test with Tukey’s post-test for normally-distributed data. *p < 0.05; ** p < 0.01 and ****p < 0.0001

**Fig. 2:**
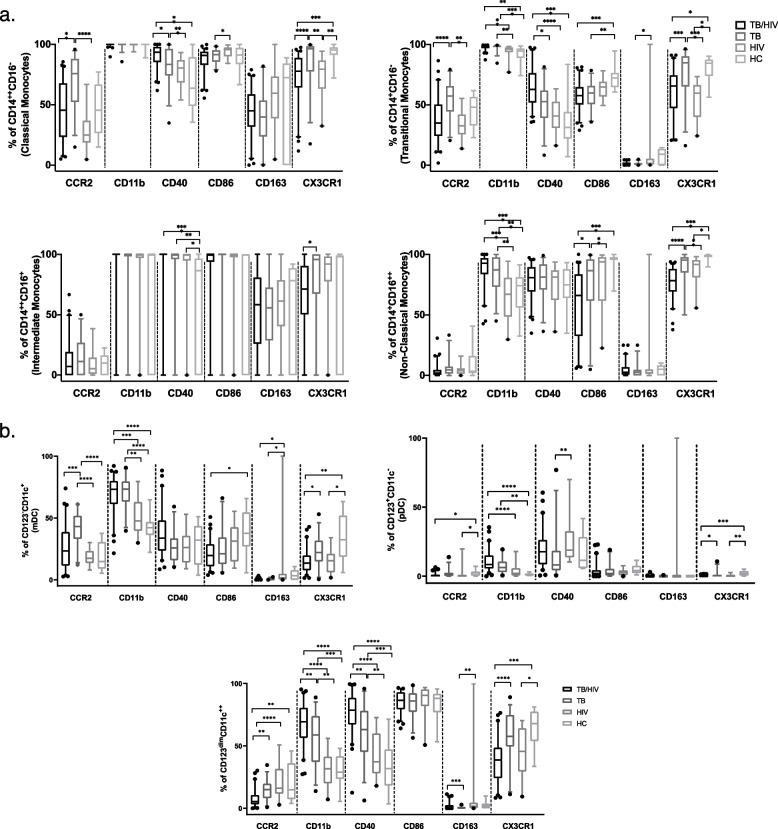
Cell surface expression of CCR2, CD11b, CD40, CD86, CD163 and CXC3R1 on monocyte (**a**) and DC (**b**) cell subsets across study groups (TB/HIV, TB, HIV and HC). Boxes in **a** and **b** represent median and interquartile ranges; whiskers represent 5–95 percentiles. Differences among the groups were assessed using a Kruskal–Wallis test with Dunn’s post-test for non-parametric data and one-way ANOVA with Tukey’s post-test for parametric data. *p < 0.05; ** p < 0.01; ***p < 0.001; and ****p < 0.0001

To determine if frequency and phenotypes of the systemic monocytes and dendritic cells are modified by TB and TB/HIV co-infection we used flow cytometry to measure and characterize monocytes and dendritic cells ex vivo in PBMCs the four groups: TB/HIV, TB, HIV and heathy controls (HC), (Figs. 1, and 2, Sup. Table 2). The frequency of total monocytes (HLA-DR^+^CD14^+^) was significantly higher among participants with TB/HIV co-infection (*p* = 0.018) and TB (*p* = 0.021) compared to healthy controls (Fig. 1b). When characterizing different monocyte subsets, the frequency of non-classical monocytes in the HIV group was higher compared to both the TB/HIV (*p* = 0.009) and TB (*p* = 0.005) groups. Similarly, the frequency of intermediate monocytes was higher in the HIV group compared to TB/HIV group (*p* = 0.042).

The frequency of total dendritic cells (HLA-DR^+^CD14^−^) was higher in the TB/HIV (*p* = 0.020) and HIV (*p* = 0.013) groups compared to healthy controls (Fig. 1c). The frequency of mDCs was higher in the TB/HIV (*p* =  < 0.0001) and TB (*p* = 0.010) groups compared to healthy controls, while the frequency of pDCs was lower in the TB/HIV (*p* = 0.003) and TB (*p* = 0.023) groups compared to healthy controls.

Given that the monocyte and dendritic cell function and phenotype are affected by TB and HIV infection [13, 25, 26] we further evaluated the expression of six phenotypic markers expressed by monocyte and dendritic cell subsets in the four study groups. We observed significant variation in cell surface marker expression across different subsets (Fig. 2, Sup. Table 2): monocytes (Fig. 2a) and DCs. We observed an increase in % CCR2 expression in the TB group compared to both the TB/HIV and HIV groups on classical and transitional monocytes and mDCs. The opposite trend was observed for CD123^dim^ CD11c^++^ cells with lower % CCR2 expression in the TB/HIV group than the other 3 groups. Frequency of CD11b was higher in the presence of active TB infection in both the TB/HIV and TB groups compared to healthy controls and the HIV group on non-classical and transitional monocytes and all three dendritic cell subsets. Frequency of CD40 on classical and transitional monocytes and CD123^dim^ CD11c^++^ cells was highest in the TB/HIV group, gradually decreasing in TB, HIV and healthy controls. Frequency of CD86 was significantly lower in the TB/HIV group compared to healthy controls on non-classical, transitional monocytes and mDCs. There was a decrease in % CX3CR1 on all monocyte and DC subsets in the presence of HIV infection, with lowest expression in the TB/HIV and HIV groups compared to the TB group and healthy controls. HIV infection was associated with an increase in frequency of CD163 compared to the TB group in transitional monocytes, mDCs and CD123^dim^ CD11c^++^.

### Associations between monocyte and dendritic cell frequencies and phenotypes at active TB and time to culture conversion

We used a Cox proportional hazards model to assess the association between frequencies of monocyte and dendritic cell subsets and their phenotypes during active TB disease on time to negative culture conversion (*n* = 90, Table 2, Sup. Table 3).

**Table 2:** Significant associations between monocyte and dendritic cell phenotypes and their surface expression markers with time to culture conversion among the total cohort

	**Bivariable**			**Multivariable**		
	**HR**	**CI**	***p*-value**	**HR**	**CI**	***p*-value**
%CD11b on NCM	1.020	1.003—1.037	**0.019**	1.017	0.999 -1.035	0.059
%CX3CR1 on NCM	0.987	0.972—1.002	0.097	0.980	0.963—0.997	**0.019**

*NCM* Non-classical monocytes

In the bivariable model, increased % of CD11b expression on non-classical monocytes was associated with shorter time to culture conversion (aHR 1.02, 95%CI: 1.002–1.0437, *p* = 0.019). Increased % of CX3CR1 expression on non-classical monocytes was associated with the longer time to culture conversion in the multivariable model (aHR: 0.987, 95%CI: 0.963–0.997, *p* = 0.019).

Next, we performed a sub-analysis of TB/HIV co-infected individuals adjusting for the effects of HIV viral load, CD4 count and ART duration (*n* = 60, Sup. Table 4). In the bivariable model, higher % CD163 (aHR 0.983, 95% CI: 0.967 – 0.999, *p* = 0.043) expression on classical monocytes was significantly associated with longer time to culture conversion, however this was not significant in the multivariable model (aHR 0.985, 95%CI: 0.967 – 1.003, *p* = 0.106). As seen in the overall cohort, increased expression of CX3CR1 (aHR 0.971, 95% CI: 0.949 – 0.994, *p* = 0.013) on non-classical monocytes significantly associated with longer time to culture conversion in the multivariable model.

### Associations between monocyte and dendritic cell frequencies and phenotypes at active TB and cavitary disease

Next, we assessed the effect of monocyte and dendritic cell frequencies and phenotypes at active TB on cavitary disease as defined by the presence of lung cavitation using logistic regression models (*n* = 90, Table 3, Sup. Table 5).

**Table 3:** Significant associations between monocyte and dendritic cell phenotypes and their surface expression markers with cavitary disease among the total cohort

	**Univariable**			**Multivariable**		
	**OR**	**CI**	***p*-value**	**OR**	**CI**	***p*-value**
% Classical Monocytes (CM)	1.069	1.004—1.138	**0.037**	1.066	0.996—1.142	0.066
%CD86 on CM	1.053	1.000—1.109	**0.049**	1.054	0.994—1.117	0.079
%CX3CR1 on CM	1.022	1.001—1.043	**0.036**	1.017	0.995—1.039	0.138
%CX3CR1 on NCM	1.039	1.006—1.073	**0.022**	1.031	0.993—1.070	0.107
% Intermediate Monocytes (IM)	4.612	1.011—21.041	**0.048**	4.693	0.912—24.156	0.064
% CD86 on IM	1.015	1.002—1.029	**0.028**	1.017	1.001—1.032	**0.033**
% Transitional Monocytes (TM)	0.946	0.899—0.995	**0.033**	0.944	0.892—0.999	**0.047**
% Dendritic cells (%HLA-DR^+^ CD14^−^)	0.946	0.894—1.000	**0.048**	0.945	0.890—1.005	0.071

In the univariable model among the total cohort, higher frequency of the following cell populations was associated with presence of lung cavitation: % classical monocytes (*p* = 0.037), %CD86 on classical monocytes (*p* = 0.049), %CX3CR1 on classical monocytes (*p* = 0.036), % CXCR31 on non-classical monocytes (*p* = 0.022), % intermediate monocytes (*p* = 0.048) and %CD86 on intermediate monocytes (*p* = 0.028). Following multivariable analysis, %CD86 on intermediate monocytes remained significantly associated with presence of lung cavitation (aOR 1.017, 95% CI: 1.001–1.032), with a trend observed for % classical monocytes (aOR 1.066, 95%CI 0.996 – 1.142, *p* = 0.066).

Frequency of transitional monocytes was associated with absence of lung cavitation in the univariable (*p* = 0.033) and multivariable analysis (aOR 0.944, 95% CI: 0.892–0.999, *p* = 0.047). Increase in total dendritic cells frequency was associated with the absence of lung cavitation [univariable model (aOR 0.946, 95%CI 0.894 – 1.000, *p* = 0.048); multivariable model (aOR 0.945, 95%CI 0.890 – 1.005, *p* = 0.071)].

Among TB/HIV co-infected individuals (Sup. Table 6), increase in %CD11b on non-classical monocytes was significantly associated with cavitary disease in both univariable (*p* = 0.017) and multivariable (aOR 1.072, 95% CI: 1.013–1.135, *p* = 0.017) models. Increase in %CD86 on intermediate monocytes was significantly associated with cavitary disease in the univariable model (*p* = 0.030), with a trend observed in the multivariable model (aOR 1.020, 95% CI: 0.999–1.040, *p* = 0.062).

## Discussion

Both TB and HIV infections have profound effects on the host immune system. Here we described differences in monocyte and dendritic cell frequencies and phenotypes in TB and HIV infection/co-infection.

The overall increase in frequency of circulating monocytes in TB and TB/HIV coinfected patients is consistent with previous studies that demonstrated an increase in circulating monocytes in TB patients [9, 27]. We observed an increase in the frequency of non-classical and intermediate monocytes in the HIV group compared to TB and TB/HIV participant groups. Non-classical and intermediate monocytes are known to be expanded in HIV positive individuals irrespective of ART treatment [28–30]. These two monocyte subsets, characterized by high CD16 expression, are generally considered pro-inflammatory, and are known to release large amounts of inflammatory cytokines and have high expression of CX3CR1 [31, 32].

HIV infection was associated with increased frequency of total dendritic cells in both TB/HIV and HIV groups compared to HCs. TB/HIV and TB participant groups had increased frequency of circulating mDCs compared to healthy controls and this was observed previously in patients with pulmonary TB [33]. In contrast, we observed lower frequency of pDCs in TB/HIV and TB groups compared to healthy controls. This decrease in peripheral pDCs is likely observed due to migration of the pDCs to the site of infection, as influx of pDCs into the lungs has been described as one of the defining features of active pulmonary TB in the macaque model [34].

We observed significant heterogeneity in measured cell surface marker expression across different cell subsets in the four study groups. We observed an increase in frequency of CCR2 on classical and transitional monocytes and mDCs in the TB group compared to TB/HIV and HIV groups. CCR2 is a chemokine receptor that drives the recruitment of cells to the inflammatory sites. Increase in CCR2 was linked with the high level of immune activation and inflammation in HIV infection [35] as well as pathogenesis of several immune-mediated diseases [36, 37]. In Mtb infection, CCR2 expression was shown to play an important role in the control of infection through changes in the recruitment of monocyte, dendritic cells and T cells into the lung [38, 39].

Mtb infection also increased CD11b expression in both TB/HIV and TB groups compared to healthy controls and HIV positive participants on non-classical, transitional monocytes and dendritic cells subsets. Integrin CD11b, a receptor for soluble intercellular adhesion molecule-1 (ICAM-1), plays an important role in inflammation and macrophage polarization [40]. We have previously identified CD11b ligand, sICAM-1 plasma levels as one of the predictors of TB recurrence in TB/HIV co-infected individuals [5]. Additionally, increased frequency of CD11b on non-classical monocytes was associated with shorter time to culture conversion among the total TB and TB/HIV IMPRESS cohort as well as presence of lung cavitation in the TB/HIV co-infected group, suggesting a role in both pathogen clearance and associated immunopathogenesis.

An increase in CD40 expression on classical and transitional monocytes and CD123^dim^ CD11c^++^ dendritic cells was observed in TB/HIV co-infected participants, gradually decreasing in TB group, followed by HIV and HCs. CD40 is a costimulatory receptor expressed on a variety of cells including monocytes, DCs and B cells, and plays an important role in T cell and macrophage activation through interaction with its ligand CD40L [41]. Upregulation of CD40 on monocyte and dendritic cells has been reported in HIV infection and is likely a consequence of increasing levels of immune activation and pro-inflammatory cytokines [42]. Mtb infection was shown to impair the CD40-CD40L interaction resulting in suboptimal antigen-specific CD4^+^ T cell immune response [43].

Expression of co-stimulatory receptor CD86 was downregulated in the TB/HIV group compared to healthy controls in transitional and non-classical monocytes as well as mDCs. Both TB and HIV infections can lead to downregulation of CD86 [11, 44, 45] which is likely to result in impaired antigen presentation and T cell activation. Additionally, we observed a significant association between increased expression of CD86 on classical and intermediate monocytes and presence of lung cavitation in the overall cohort implicating it in TB immunopathogenesis.

Presence of HIV infection resulted in decreased frequency of CX3CR1 in the HIV and TB/HIV groups compared to TB group and healthy controls on all monocyte and dendritic cell subsets. HIV Tat was previously shown to supress CX3CR1 expression at both mRNA and protein level with subsequent induction of proinflammatory cytokines [46]. Decreased expression of CX3CR1 on circulating monocytes was also identified as a feature of sepsis-induced immunosuppression [47], and this decrease in expression in highly inflammatory conditions could result in impairment of cell migration into the tissues and negatively impact pathogen clearance. This is supported by the observed association between CX3CR1 expression on non-classical monocytes and longer time to culture conversion and presence of lung cavitation.

HIV infection was associated with increased frequency of CD163 on transitional monocytes, mDCs and CD123^dim^ CD11c^++^ cells and increase in % CD163 on classical monocytes was associated with longer time to culture conversion in the HIV co-infected group. CD163 is a scavenger receptor expressed on cells of monocyte and macrophages lineage [48, 49]. HIV infection was previously associated with increased frequency of CD163 + monocytes and this increase was associated with decrease in CD4^+^ T cells and increase in viral loads [50, 51]. Additionally, CD163 has been previously proposed as a marker to monitor disease progression and treatment efficacy in TB disease [52].

There are several limitations to our study, including a relatively small sample size especially in the heathy control group and subsequent inability to control for clinical and demographic variables that could contribute to the observed differences in innate immune cell phenotypes. Information on the presence of latent TB in the HIV cohort was not available. Additionally, cryopreservation can affect cell phenotype and function [53]. Here we are analyzing systemic cell populations that may differ significantly from cell populations observed in the lungs, at the site of Mtb replication. Nevertheless, our data contributes to a deeper understanding of the host immune changes during TB/HIV co-infection and its impact on TB disease pathogenesis. A better understanding of the HIV and TB mediated immune changes and their effects on disease outcome could lead to the discovery of diagnostic biomarkers and novel disease targets for host-directed therapies to reduce the burden of both diseases in vulnerable populations. Modulation of host immune pathways that impact disease pathology could limit Mtb replication and improve disease outcomes without concerns for antimicrobial resistance.

## Supplementary Information

**Additional file 1: Supplementary Table 1.** Monocyte and Dendritic cell Phenotyping Antibodies. **Supplementary Figure 1.** Representative parent gating. The staining profile of PBMC sample from a TB/HIV participant is shown. **Supplementary Figure 2.** Expression of CD11b, CD163, CD86, CD40, CCR2 and CXCR31 on classical monocytes (A) from a representative TB/HIV participant B) FMO controls. **Supplementary Table 2.** Cell population frequencies among different study groups. **Supplementary Table 3.** Association between monocyte and dendritic cell phenotypes and their surface expression markers with overall time to culture conversion among the total cohort. **Supplementary Table 4.** Association between monocyte and dendritic cell phenotypes and their surface expression markers with overall time to culture conversion among the HIV positive individuals. **Supplementary Table 5.** Association between monocyte and dendritic cell phenotypes and their surface expression markers with cavitary disease among the total cohort. **Supplementary Table 6.** Association between monocyte and dendritic cell phenotypes and their surface expression markers with cavitary disease among the HIV positive individuals.

## Data Availability

The datasets generated and analysed during the current study are available from the corresponding author on reasonable request.

## Abbreviations

CAPRISA: Centre for the AIDS Programme of Research in South Africa
IMPRESS: Improving Retreatment Success
TB: Tuberculosis
Mtb: *Mycobacterium tuberculosis*
HIV: Human Immunodeficiency Virus
DCs: Dendritic cells
mDCs: Myeloid dendritic cells
pDCs: Plasmacytoid cells
HC: Healthy controls
PBMCs: Peripheral blood mononuclear cells
CM: Classical monocytes
IM: Intermediate monocytes
NCM: Non-classical monocytes
TM: Transitional monocytes
ICAM: Intercellular adhesion molecule
ART: Antiretroviral therapy
OR: Odds ratio
aOR: Adjusted odds ratio
HR: Hazard ratio
aHR: Adjusted hazard ratio
BMI: Body mass index
IQR: Interquartile range
KZN: KwaZulu-Natal
SA: South Africa
PCZCDC: Prince Cyril Zulu Communicable Disease Centre
PLWH: People living with HIV
ACD: Acid citrate dextrose
DMSO: Dimethyl sulfoxide
FBS: Fetal bovine serum
