# Supplementary material for: Blood monocyte and dendritic cell profiles among people living with HIV with Mycobacterium tuberculosis co-infection
Source: BMC Immunol. 2023 Jul 21;24:21. doi: 10.1186/s12865-023-00558-z (PMC10362598; doi:10.1186/s12865-023-00558-z)
Supplement: Supplementary file 1 — Additional file 1: Supplementary Table 1. Monocyte and Dendritic cell Phenotyping Antibodies. Supplementary Figure 1. Representative parent gating. The staining profile of PBMC sample from a TB/HIV participant is shown. Supplementary Figure 2. Expression of CD11b, CD163, CD86, CD40, CCR2 and CXCR31 on classical monocytes (A) from a representative TB/HIV participant B) FMO controls. Supplementary Table 2. Cell population frequencies among different study groups. Supplementary Table 3. Association between monocyte and dendritic cell phenotypes and their surface expression markers with overall time to culture conversion among the total cohort. Supplementary Table 4. Association between monocyte and dendritic cell phenotypes and their surface expression markers with overall time to culture conversion among the HIV positive individuals. Supplementary Table 5. Association between monocyte and dendritic cell phenotypes and their surface expression markers with cavitary disease among the total cohort. Supplementary Table 6. Association between monocyte and dendritic cell phenotypes and their surface expression markers with cavitary disease among the HIV positive individuals. [file 12865_2023_558_MOESM1_ESM.docx]

**Supplementary material**

| **Supplementary Table 1.** Monocyte and Dendritic cell Phenotyping Antibodies   \| **Antibody** \| **Fluorochrome** \| **Clone** \| **Cat #** \| **Manufacturer** \| **Volume (µl) per test (50µl)** \| \| --- \| --- \| --- \| --- \| --- \| --- \| \| CD11c \| PECy5 \| 3.9 \| 301610 \| BioLegend \| 1 \| \| HLA-DR \| PE CF594 \| G46-6 \| 562304 \| BD \| 1 \| \| CD3 \| Alexa Fluor 700 \| SK7 \| 344822 \| BioLegend \| 1 \| \| CD56 \| Alexa Fluor 700 \| 5.1 H11 \| 362522 \| BioLegend \| 2 \| \| CD19 \| Alexa Fluor 700 \| HIB1 \| 302226 \| BioLegend \| 2 \| \| CD14 \| APC Cy7 \| HCD14 \| 325620 \| BioLegend \| 1 \| \| CD123 \| BV421 \| 6H6 \| 48-1239-42 \| eBioscience \| 2 \| \| CD16 \| BV786 \| 3G8 \| 302046 \| BioLegend \| 1 \| \| CD86 \| BV650 \| IT2.2 \| 305428 \| BioLegend \| 2.5 \| \| CD40 \| BV711 \| 5C3 \| 334334 \| BioLegend \| 2 \| \| CD163 \| PE \| GHI/61 \| 333606 \| BioLegend \| 1 \| \| CD11b \| PECy7 \| ICRF44 \| 301322 \| BioLegend \| 1 \| \| CX3CR1 \| PerCP Cy5.5 \| 2A9-1 \| 341614 \| BioLegend \| 2 \| \| CCR2 - CD192 \| Alex Fluor 647 \| 48607 \| 558406 \| BD \| 10 \| \| Live/Dead \| AmCyan \|  \| L34957 \| ThermoFisher \| 3* \| |
| --- | --- | --- | --- | --- | --- | --- | --- | --- | --- | --- | --- | --- | --- | --- | --- | --- | --- | --- | --- | --- | --- | --- | --- | --- | --- | --- | --- | --- | --- | --- | --- | --- | --- | --- | --- | --- | --- | --- | --- | --- | --- | --- | --- | --- | --- | --- | --- | --- | --- | --- | --- | --- | --- | --- | --- | --- | --- | --- | --- | --- | --- | --- | --- | --- | --- | --- | --- | --- | --- | --- | --- | --- | --- | --- | --- | --- | --- | --- | --- | --- | --- | --- | --- | --- | --- | --- | --- | --- | --- | --- | --- | --- | --- | --- | --- | --- |

*3µl of the following dilution: 2µl of stock in 80µl of sdH20


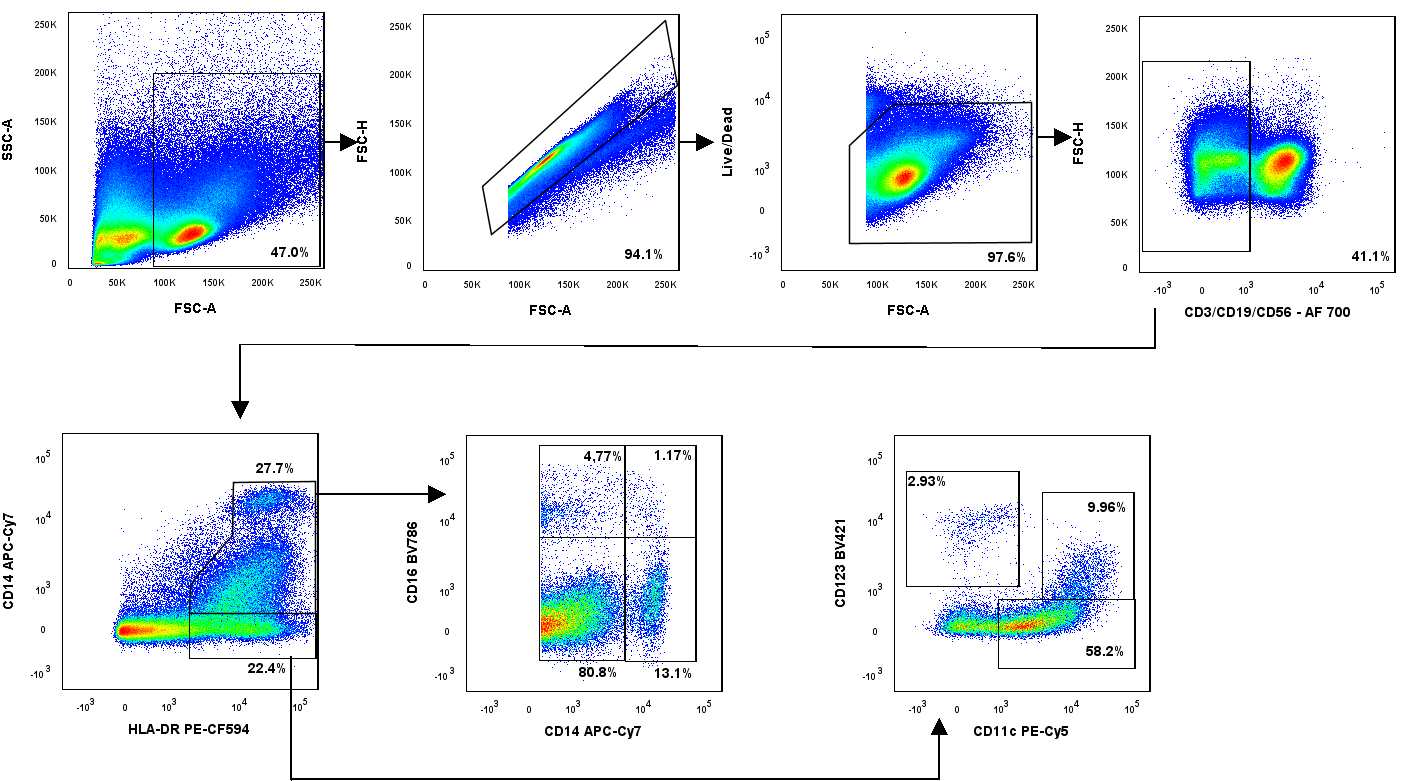


**Supplementary Figure 1.** Representative parent gating. The staining profile of PBMC sample from a TB/HIV participant is shown.


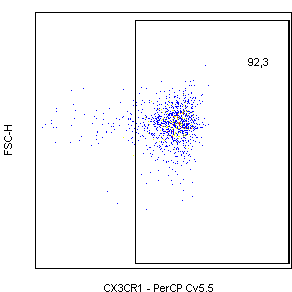

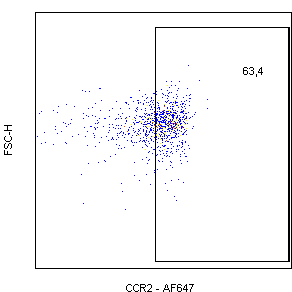

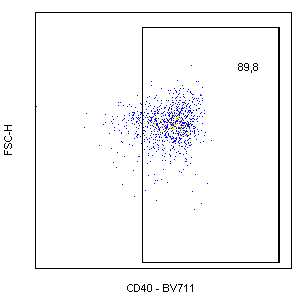

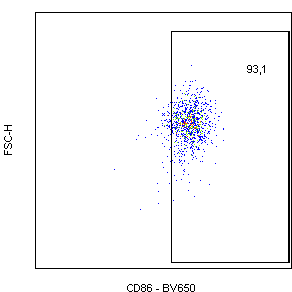

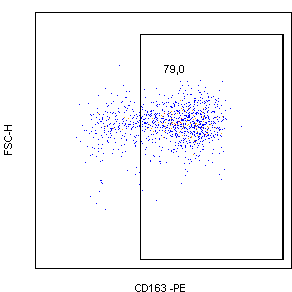

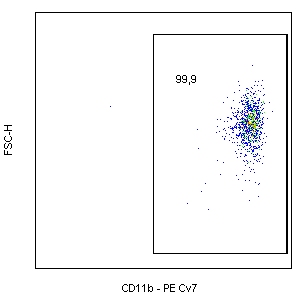

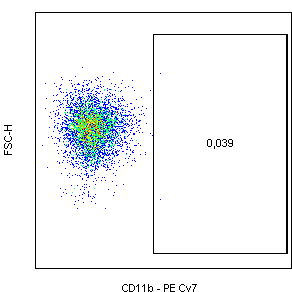


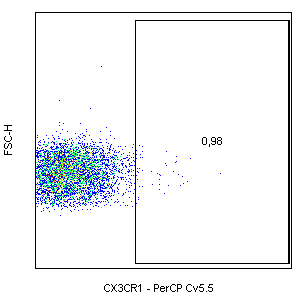

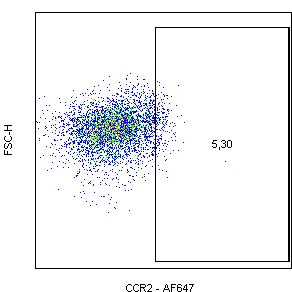

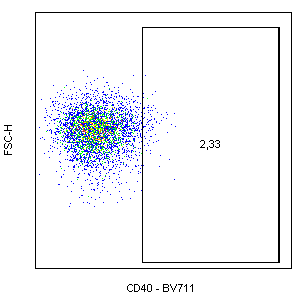

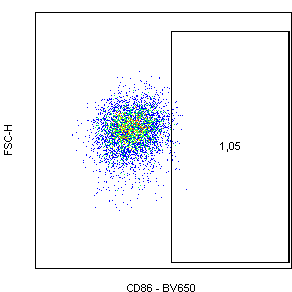

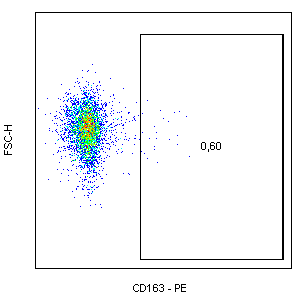


**Supplementary Figure 2.** Expression of CD11b, CD163, CD86, CD40, CCR2 and CXCR31 on classical monocytes (A) from a representative TB/HIV participant B) FMO controls

**Supplementary Table 2.** Cell population frequencies among different study groups

|  | **TB/HIV** | |  | **TB** | |  | **HIV** | |  | **HC** | | **p-value** |
| --- | --- | --- | --- | --- | --- | --- | --- | --- | --- | --- | --- | --- |
|  | **N** | **Median (IQR)** |  | **N** | **Median (IQR)** |  | **N** | **Median (IQR)** |  | **N** | **Median (IQR)** |  |
| **% CD14 Positive (HLA-DR+ CD14+)** | 60 | 36.10 (29.08 – 45.53) |  | 30 | 36.35 (30.78 – 42.18) |  | 19 | 35.00 (26.20 – 48.80) |  | 11 | 25.30 (17.70 – 35.00) | **0.0213** |
|  |  |  |  |  |  |  |  |  |  |  |  |  |
| **% Classical Monocytes (CM)** |  | 10.60 (4.38 – 14.35) |  |  | 10.55 (3.44 – 10.58) |  |  | 25.70 (4.06 – 37.20) |  |  | 59.70 (0.40 – 64.90) | **0.0447** |
| % CCR2 |  | 45.50 (23.40 – 57.80) |  |  | 75.85 (52.38 – 87.98) |  |  | 25.00 (18.70 – 36.80) |  |  | 45.50 (26.60 – 66.70) | **<0.0001** |
| % CD11b |  | 100.00 (99.90 – 100.00) |  |  | 100.00 (99.90 – 100.00) |  |  | 100.00 (99.60 – 100.00) |  |  | 100.00 (99.80 – 100.00) | 0.6012 |
| % CD40 |  | 93.55 (85.40 – 97.83) |  |  | 83.50 (74.23 – 96.15) |  |  | 80.40 (68.40 – 87.00) |  |  | 63.80 (49.00 – 89.40) | **<0.0001** |
| % CD86 |  | 90.90 (83.13 – 93.98) |  |  | 91.50 (85.70 – 95.15) |  |  | 96.70 (90.20 – 97.40) |  |  | 91.20 (83.70 – 97.10) | **0.0285** |
| % CD163 |  | 44.95 (31.85 – 58.70) |  |  | 39.85 (23.68 – 53.40) |  |  | 59.50 (38.80 – 73.40) |  |  | 72.50 (0 – 84.10) | 0.0927 |
| % CX3CR1 |  | 77.85 (64.30 – 88.75) |  |  | 96.35 (77.60 – 98.33) |  |  | 79.50 (64.30 – 86.70) |  |  | 95.30 (91.30 – 97.60) | **<0.0001** |
|  |  |  |  |  |  |  |  |  |  |  |  |  |
| **% Non -Classical monocytes (NCM)** |  | 1.45 (0.67 – 3.43) |  |  | 1.05 (0.53 – 3.52) |  |  | 4.85 (1.72 – 10.30) |  |  | 5.42 (0.55 – 8.69) | **0.0019** |
| % CCR2 |  | 1.92 (0.65 – 4.51) |  |  | 4.65 (1.52 – 7.21) |  |  | 3.96 (1.37 – 5.74) |  |  | 3.38 (2.10 – 16.00) | 0.1062 |
| % CD11b |  | 92.70 (83.70 – 96.78) |  |  | 87.15 (73.23 – 97.58) |  |  | 67.00 (48.60 – 80.60) |  |  | 74.10 (56.00 – 81.20) | **<0.0001** |
| % CD40 |  | 80.75 (69.30 – 89.38) |  |  | 81.30 (70.95 – 91.70) |  |  | 81.30 (62.40 – 86.80) |  |  | 74.80 (64.00 – 87.80) | 0.6320 |
| % CD86 |  | 66.15 (32.70 – 83.43) |  |  | 86.50 (61.75 – 95.90) |  |  | 93.90 (61.70 – 97.60) |  |  | 96.90 (95.50 – 97.70) | **<0.0001** |
| % CD163 |  | 2.68 (0.66 – 6.76) |  |  | 2.55 (0.00 – 4.59) |  |  | 2.37 (1.36 – 5.00) |  |  | 5.07 (0.00 – 8.25) | 0.7701 |
| % CX3CR1 |  | 78.30 (69.30 – 87.93) |  |  | 95.25 (85.03 – 97.63) |  |  | 91.70 (81.10 – 95.20) |  |  | 99.00 (97.80 – 99.60) | **<0.0001** |
|  |  |  |  |  |  |  |  |  |  |  |  |  |
| **% Intermediate monocytes (IM)** |  | 0.08 (0.03 – 0.53) |  |  | 0.19 (0.02 – 0.82) |  |  | 0.51 (0.18 – 1.94) |  |  | 1.78 (0.00 – 2.36) | **0.0216** |
| % CCR2 |  | 7.14 (0.00 – 19.18) |  |  | 11.25 (0.00 – 26.80) |  |  | 5.26 (1.120 – 14.40) |  |  | 9.93 (0.00 – 16.40) | 0.6318 |
| % CD11b |  | 100.00 (100.00 – 100.00) |  |  | 100.00 (98.48 – 100.00) |  |  | 100.00 (97.10 – 100.00) |  |  | 100.00 (0.00 – 100.00) | **0.0282** |
| % CD40 |  | 100.00 (100.00 – 100.00) |  |  | 100.00 (95.98 – 100.00) |  |  | 99.80 (95.00 – 100.00) |  |  | 86.60 (0.00 – 96.50) | **<0.0001** |
| % CD86 |  | 100.00 (93.90 – 100.00) |  |  | 100.00 (99.75 – 100.00) |  |  | 100.00 (97.70 – 100.00) |  |  | 99.60 (0.00 – 100.00) | 0.4167 |
| % CD163 |  | 58.30 (25.90 – 80.68) |  |  | 55.70 (28.75 – 72.53) |  |  | 61.30 (40.50 – 78.70) |  |  | 78.20 (0 – 88.50) | 0.7277 |
| % CX3CR1 |  | 71.35 (50.25 – 90.43) |  |  | 95.85 (67.45 – 100) |  |  | 91.90 (77.70 – 98.10) |  |  | 98.20 (0 – 99.00) | **0.0087** |
|  |  |  |  |  |  |  |  |  |  |  |  |  |
| **%Transitional monocytes (TM)** |  | 87.25 (79.85 – 92.90) |  |  | 88.20 (77.13 – 96.08) |  |  | 72.60 (49.50 – 88.50) |  |  | 32.70 (22.30 – 98.90) | **0.0253** |
| % CCR2 |  | 35.05 (24.33 – 50.30) |  |  | 57.25 (44.58 – 65.50) |  |  | 32.70 (25.60 – 41.90) |  |  | 48.10 (32.90 – 56.50) | **<0.0001** |
| % CD11b |  | 98.15 (97.00 – 98.70) |  |  | 98.55 (97.90 – 99.03) |  |  | 96.20 (93.70 – 97.50) |  |  | 94.60 (88.90 – 96.60) | **<0.0001** |
| % CD40 |  | 62.80 (52.03 – 76.35) |  |  | 52.60 (39.20 – 61.95) |  |  | 40.70 (31.00 – 52.60) |  |  | 31.30 (22.10 – 44.10) | **<0.0001** |
| % CD86 |  | 57.60 (50.35 – 64.75) |  |  | 59.85 (50.60 – 65.73) |  |  | 64.80 (57.00 – 69.10) |  |  | 72.20 (64.80 – 76.50) | **0.0002** |
| % CD163 |  | 1.50 (0.82 – 2.18) |  |  | 1.01 (0.33 – 1.68) |  |  | 1.70 (0.95 – 5.31) |  |  | 9.08 (0.13 – 13.50) | **0.0134** |
| % CX3CR1 |  | 65.60 (47.38 – 74.48) |  |  | 84.85 (65.20 – 90.15) |  |  | 59.70 (40.00 – 66.30) |  |  | 84.50 (74.10 – 87.40) | **<0.0001** |
|  |  |  |  |  |  |  |  |  |  |  |  |  |
| **% CD14 Negative (HLA-DR+ CD14-)** |  | 31.45 (24.70 – 36.83) |  |  | 28.65 (21.38 – 39.05) |  |  | 31.80 (28.10 – 38.60) |  |  | 16.30 (14.10 – 34.90) | **0.0147** |
| **% mDC** |  | 46.15 (35.40 – 55.08) |  |  | 38.10 (24.78 – 51.05) |  |  | 29.70 (24.20 – 41.60) |  |  | 21.50 (17.90 – 31.10) | **<0.0001** |
| % CCR2 |  | 23.30 (11.55 – 38.50) |  |  | 43.15 (33.25 – 51.70) |  |  | 17.30 (13.70 – 23.30) |  |  | 14.80 (9.09 – 30.50) | **<0.0001** |
| % CD11b |  | 73.20 (61.33 – 79.78) |  |  | 73.40 (63.35 – 79.53) |  |  | 47.70 (39.70 – 62.90) |  |  | 42.10 (36.20 – 46.70) | **<0.0001** |
| % CD40 |  | 33.70 (23.65 – 47.93) |  |  | 25.80 (15.80 – 33.40) |  |  | 26.00 (14.10 – 35.50) |  |  | 32.10 (12.30 – 43.50) | **0.0244** |
| % CD86 |  | 19.55 (10.98 – 28.75) |  |  | 21.05 (12.75 – 34.20) |  |  | 31.20 (15.00 – 42.50) |  |  | 37.60 (27.10 – 54.40) | **0.0033** |
| % CD163 |  | 0.37 (0.16 – 0.67) |  |  | 0.24 (0.13 – 0.92) |  |  | 1.67 (0.42 – 4.67) |  |  | 3.31 (0.03 – 7.69) | **0.0064** |
| % CX3CR1 |  | 13.45 (7.88 – 19.55) |  |  | 22.10 (14.85 – 31.60) |  |  | 15.40 (6.22– 20.90) |  |  | 32.40 (18.70 – 51.90) | **0.0003** |
|  |  |  |  |  |  |  |  |  |  |  |  |  |
| **% CD123^dim^CD11c^++^** |  | 8.00 (3.48 – 11.83) |  |  | 11.10 (7.41 – 27.43) |  |  | 8.50 (2.80 – 20.00) |  |  | 5.58 (3.38 – 11.10) | 0.0865 |
| % CCR2 |  | 5.39 (3.230 – 10.48) |  |  | 15.00 (8.21 – 19.60) |  |  | 16.10 (11.80 – 31.60) |  |  | 14.90 (7.48 – 36.00) | **<0.0001** |
| % CD11b |  | 69.30 (56.60 – 80.55) |  |  | 58.75 (37.28 – 73.88) |  |  | 31.70 (20.20 – 41.30) |  |  | 28.90 (23.70 – 41.70) | **<0.0001** |
| % CD40 |  | 78.65 (67.33 – 88.50) |  |  | 63.10 (45.35 – 78.05) |  |  | 37.30 (28.10 –59.90) |  |  | 31.80 (18.30 – 47.10) | **<0.0001** |
| % CD86 |  | 86.50 (79.53 – 93.00) |  |  | 86.10 (77.48 – 92.25) |  |  | 90.50 (82.00 – 95.20) |  |  | 88.10 (77.10 – 91.70) | 0.6115 |
| % CD163 |  | 1.10 (0.43 – 2.46) |  |  | 0.24 (0.05 – 0.81) |  |  | 1.75 (0.28 – 4.37) |  |  | 1.92 (0.00 – 3.74) | **0.0002** |
| % CX3CR1 |  | 38.70 (24.28 – 48.50) |  |  | 57.60 (49.58 – 74.78) |  |  | 45.70 (29.60 – 64.10) |  |  | 68.00 (54.00 – 77.50) | **<0.0001** |
|  |  |  |  |  |  |  |  |  |  |  |  |  |
| **% pDC** |  | 2.13 (0.97 – 3.14) |  |  | 2.01 (1.21 – 3.86) |  |  | 2.40 (1.12 – 3.40) |  |  | 4.39 (2.69 – 7.80) | **0.0067** |
| % CCR2 |  | 0.36 (0.00 – 1.21) |  |  | 1.05 (0.31 – 2.35) |  |  | 0.25 (0.00 – 0.88) |  |  | 2.37 (0.36 – 3.35) | **0.0024** |
| % CD11b |  | 8.40 (5.66 – 15.00) |  |  | 6.21 (2.96 – 10.58) |  |  | 2.05 (0.57 – 5.73) |  |  | 1.12 (0.47 – 1.94) | **<0.0001** |
| % CD40 |  | 17.75 (8.65 – 26.18) |  |  | 8.10 (4.39 – 18.43) |  |  | 18.80 (13.70 – 32.60) |  |  | 11.50 (5.91 – 28.40) | **0.0117** |
| % CD86 |  | 2.07 (0.96 – 4.23) |  |  | 1.86 (0.55 – 5.29) |  |  | 2.75 (0.78 – 4.13) |  |  | 4.04 (2.38 – 7.43) | 0.2319 |
| % CD163 |  | 0.00 (0.00-0.00) |  |  | 0.00 (0.00-0.00) |  |  | 0.00 (0.00-0.00) |  |  | 0.00 (0.00-0.00) | 0.6551 |
| % CX3CR1 |  | 0.00 (0.00 – 0.29) |  |  | 0.30 (0.00 – 0.92) |  |  | 0.00 (0.00 – 0.78) |  |  | 1.90 (0.45 – 3.77) | **<0.0001** |
|  |  |  |  |  |  |  |  |  |  |  |  |  |

**Supplementary Table 3.** Association between monocyte and dendritic cell phenotypes and their surface expression markers with overall time to culture conversion among the total cohort.

|  | **Bivariable** | | |  | **Multivariable** | | |  |
| --- | --- | --- | --- | --- | --- | --- | --- | --- |
|  | **HR** | **CI** | **p-value** |  | **HR** | **CI** | **p-value** | **q-value** |
| **% CD14 Positive (HLA-DR+ CD14+)** | 0.992 | 0.974 - 1.010 | 0.359 |  | 0.981 | 0.961 - 1.002 | 0.078 | 0.898 |
| **% Classical Monocytes (CM)** | 0.989 | 0.966 - 1.013 | 0.376 |  | 1.002 | 0.977 - 1.027 | 0.903 | 0.968 |
| % CCR2 | 0.994 | 0.986 - 1.003 | 0.184 |  | 0.996 | 0.987 - 1.006 | 0.441 | 0.898 |
| % CD11b | 0.951 | 0.850 - 1.063 | 0.377 |  | 0.965 | 0.860 - 1.082 | 0.537 | 0.898 |
| % CD40 | 0.999 | 0.980 - 1.018 | 0.891 |  | 1.001 | 0.978 - 1.024 | 0.958 | 0.968 |
| % CD86 | 0.989 | 0.964 - 1.013 | 0.361 |  | 1.013 | 0.984 - 1.043 | 0.396 | 0.898 |
| % CD163 | 0.995 | 0.984 - 1.006 | 0.358 |  | 0.995 | 0.983 - 1.007 | 0.416 | 0.898 |
| % CX3CR1 | 0.996 | 0.987 - 1.005 | 0.400 |  | 0.998 | 0.987 - 1.009 | 0.717 | 0.914 |
|  |  |  |  |  |  |  |  |  |
| **% Non-Classical Monocytes (NCM)** | 0.976 | 0.916 - 1.040 | 0.460 |  | 0.986 | 0.928 - 1.047 | 0.646 | 0.914 |
| % CCR2 | 0.993 | 0.964 - 1.023 | 0.643 |  | 0.988 | 0.952 - 1.025 | 0.518 | 0.898 |
| % CD11b | 1.020 | 1.003 - 1.037 | **0.019** |  | 1.017 | 0.999 - 1.035 | 0.059 | 0.898 |
| % CD40 | 1.000 | 0.985 - 1.016 | 0.970 |  | 1.007 | 0.989 - 1.026 | 0.427 | 0.898 |
| % CD86 | 0.999 | 0.992 - 1.006 | 0.755 |  | 1.001 | 0.994 - 1.009 | 0.712 | 0.914 |
| % CD163 | 1.014 | 0.975 - 1.054 | 0.491 |  | 1.032 | 0.990 - 1.077 | 0.141 | 0.898 |
| % CX3CR1 | 0.987 | 0.972 - 1.002 | 0.097 |  | 0.980 | 0.963 - 0.997 | **0.019** | 0.898 |
|  |  |  |  |  |  |  |  |  |
| **% Intermediate Monocytes (IM)** | 0.881 | 0.662 - 1.171 | 0.382 |  | 0.897 | 0.678 - 1.188 | 0.449 | 0.898 |
| % CCR2 | 1.006 | 0.993 - 1.013 | 0.361 |  | 1.008 | 0.994 - 1.023 | 0.240 | 0.898 |
| % CD11b | 1.001 | 0.994 - 1.008 | 0.789 |  | 1.003 | 0.995 - 1.011 | 0.448 | 0.898 |
| % CD40 | 1.001 | 0.994 - 1.009 | 0.723 |  | 1.004 | 0.995 - 1.012 | 0.374 | 0.898 |
| % CD86 | 1.001 | 0.995 - 1.008 | 0.729 |  | 1.003 | 0.996 - 1.010 | 0.406 | 0.898 |
| % CD163 | 0.997 | 0.990 - 1.003 | 0.313 |  | 0.998 | 0.991 - 1.005 | 0.581 | 0.898 |
| % CX3CR1 | 1.002 | 0.996 - 1.009 | 0.512 |  | 1.004 | 0.997 - 1.001 | 0.229 | 0.898 |
|  |  |  |  |  |  |  |  |  |
| **% Transitional Monocytes (TM)** | 1.009 | 0.991 - 1.027 | 0.348 |  | 1.001 | 0.983 - 1.020 | 0.902 | 0.968 |
| % CCR2 | 0.993 | 0.981 - 1.004 | 0.218 |  | 0.99 | 0.977 - 1.004 | 0.158 | 0.898 |
| % CD11b | 0.955 | 0.867 - 1.052 | 0.353 |  | 0.943 | 0.856 - 1.040 | 0.241 | 0.898 |
| % CD40 | 1.000 | 0.987 - 1.013 | 0.977 |  | 1.003 | 0.988 - 1.017 | 0.716 | 0.914 |
| % CD86 | 1.004 | 0.984 - 1.025 | 0.668 |  | 1.014 | 0.994 - 1.035 | 0.169 | 0.898 |
| % CD163 | 0.958 | 0.787 - 1.166 | 0.669 |  | 1.079 | 0.872 - 1.334 | 0.485 | 0.898 |
| % CX3CR1 | 0.995 | 0.984 - 1.006 | 0.392 |  | 0.994 | 0.981 - 1.007 | 0.371 | 0.898 |
|  |  |  |  |  |  |  |  |  |
| **% CD14 Negative (HLA-DR+ CD14-)** | 1.009 | 0.984 - 1.036 | 0.483 |  | 1.004 | 0.978 - 1.030 | 0.787 | 0.968 |
| **% mDC** | 1.004 | 0.991 - 1.017 | 0.565 |  | 1.001 | 0.985 - 1.017 | 0.908 | 0.968 |
| % CCR2 | 0.994 | 0.982 - 1.006 | 0.300 |  | 0.987 | 0.973 - 1.002 | 0.091 | 0.898 |
| % CD11b | 1.001 | 0.987 - 1.015 | 0.894 |  | 0.991 | 0.975 - 1.008 | 0.314 | 0.898 |
| % CD40 | 1.000 | 0.987 - 1.012 | 0.945 |  | 1.000 | 0.986 - 1.015 | 0.968 | 0.968 |
| % CD86 | 0.997 | 0.981 - 1.013 | 0.691 |  | 1.001 | 0.983 - 1.020 | 0.905 | 0.968 |
| % CD163 | 1.078 | 0.683 - 1.703 | 0.746 |  | 1.089 | 0.715 - 1.659 | 0.692 | 0.914 |
| % CX3CR1 | 0.999 | 0.976 - 1.023 | 0.932 |  | 1.007 | 0.981 - 1.033 | 0.609 | 0.914 |
|  |  |  |  |  |  |  |  |  |
| **% CD123^dim^ CD11c^++^** | 0.997 | 0.978 - 1.015 | 0.715 |  | 0.994 | 0.972 - 1.016 | 0.581 | 0.898 |
| % CCR2 | 1.000 | 0.972 - 1.028 | 0.992 |  | 0.989 | 0.957 - 1.022 | 0.510 | 0.898 |
| % CD11b | 1.003 | 0.992 - 1.014 | 0.569 |  | 1.000 | 0.987 - 1.012 | 0.940 | 0.968 |
| % CD40 | 1.000 | 0.989 - 1.011 | 0.931 |  | 0.999 | 0.986 - 1.013 | 0.934 | 0.968 |
| % CD86 | 1.000 | 0.977 - 1.023 | 0.991 |  | 1.009 | 0.982 - 1.037 | 0.498 | 0.898 |
| % CD163 | 1.068 | 0.968 - 1.177 | 0.188 |  | 1.038 | 0.936 - 1.152 | 0.477 | 0.898 |
| % CX3CR1 | 0.995 | 0.983 - 1.006 | 0.358 |  | 0.994 | 0.980 - 1.008 | 0.430 | 0.898 |
|  |  |  |  |  |  |  |  |  |
| **% pDC** | 1.064 | 0.939 - 1.206 | 0.328 |  | 1.090 | 0.947 - 1.256 | 0.231 | 0.898 |
| % CCR2 | 0.967 | 0.890 - 1.051 | 0.432 |  | 0.972 | 0.881 - 1.072 | 0.568 | 0.898 |
| % CD11b | 1.012 | 0.983 - 1.041 | 0.421 |  | 1.012 | 0.980 - 1.045 | 0.476 | 0.898 |
| % CD40 | 1.000 | 0.986 - 1.015 | 0.967 |  | 0.997 | 0.982 - 1.013 | 0.713 | 0.914 |
| % CD86 | 0.992 | 0.951 - 1.034 | 0.692 |  | 1.002 | 0.960 - 1.047 | 0.915 | 0.968 |
| % CD163 | 1.247 | 0.484 - 3.215 | 0.648 |  | 1.716 | 0.744 - 3.960 | 0.206 | 0.898 |
| % CX3CR1 | 0.972 | 0.837 - 1.129 | 0.713 |  | 0.992 | 0.845 - 1.164 | 0.918 | 0.968 |
|  |  |  |  |  |  |  |  |  |

**Supplementary Table 4.** Association between monocyte and dendritic cell phenotypes and their surface expression markers with overall time to culture conversion among the HIV positive individuals.

|  | **Bivariable** | | |  | **Multivariable** | | |  |
| --- | --- | --- | --- | --- | --- | --- | --- | --- |
|  | **HR** | **CI** | **p-value** |  | **HR** | **CI** | **p-value** | **q-value** |
| **% CD14 Positive (HLA-DR+ CD14+)** | 0.992 | 0.973 - 1.012 | 0.433 |  | 0.998 | 0.960 – 1.018 | 0.435 | 0.967 |
| **% Classical Monocytes (CM)** | 0.986 | 0.952 - 1.021 | 0.420 |  | 1.000 | 0.961 – 1.041 | 0.995 | 0.995 |
| % CCR2 | 0.997 | 0.986 - 1.009 | 0.636 |  | 0.998 | 0.985 - 1.010 | 0.709 | 0.967 |
| % CD11b | 0.962 | 0.791 - 1.170 | 0.699 |  | 0.970 | 0.800 - 1.176 | 0.756 | 0.967 |
| % CD40 | 0.995 | 0.965 - 1.025 | 0.733 |  | 1.018 | 0.981 - 1.056 | 0.349 | 0.967 |
| % CD86 | 0.993 | 0.966 - 1.020 | 0.602 |  | 1.014 | 0.975 - 1.053 | 0.493 | 0.967 |
| % CD163 | 0.983 | 0.967 - 0.999 | **0.043** |  | 0.985 | 0.967 - 1.003 | 0.106 | 0.967 |
| % CX3CR1 | 1.002 | 0.990 - 1.014 | 0.729 |  | 0.997 | 0.982 - 1.012 | 0.647 | 0.967 |
|  |  |  |  |  |  |  |  |  |
| **% Non-Classical Monocytes (NCM)** | 0.974 | 0.891 - 1.064 | 0.557 |  | 0.948 | 0.868 - 1.036 | 0.239 | 0.967 |
| % CCR2 | 1.013 | 0.962 - 1.066 | 0.636 |  | 0.991 | 0.928 - 1.059 | 0.796 | 0.967 |
| % CD11b | 1.022 | 0.999 - 1.045 | 0.059 |  | 1.016 | 0.992 - 1.040 | 0.196 | 0.967 |
| % CD40 | 1.000 | 0.981 - 1.020 | 0.966 |  | 0.999 | 0.978 - 1.022 | 0.960 | 0.992 |
| % CD86 | 0.999 | 0.991 - 1.008 | 0.881 |  | 0.996 | 0.986 - 1.006 | 0.409 | 0.967 |
| % CD163 | 1.003 | 0.957 - 1.052 | 0.891 |  | 1.006 | 0.945 - 1.071 | 0.851 | 0.967 |
| % CX3CR1 | 0.981 | 0.960 - 1.003 | 0.084 |  | 0.971 | 0.949 - 0.994 | **0.013** | 0.663 |
|  |  |  |  |  |  |  |  |  |
| **% Intermediate Monocytes (IM)** | 0.835 | 0.452 - 1.540 | 0.563 |  | 0.687 | 0.352 - 1.343 | 0.271 | 0.967 |
| % CCR2 | 1.003 | 0.988 - 1.019 | 0.689 |  | 0.999 | 0.979 - 1.018 | 0.891 | 0.967 |
| % CD11b | 0.992 | 0.981 - 1.003 | 0.147 |  | 0.997 | 0.985 - 1.010 | 0.682 | 0.967 |
| % CD40 | 0.992 | 0.981 - 1.003 | 0.147 |  | 0.998 | 0.985 - 1.011 | 0.781 | 0.967 |
| % CD86 | 0.997 | 0.989 - 1.006 | 0.530 |  | 0.998 | 0.989 - 1.008 | 0.707 | 0.967 |
| % CD163 | 0.992 | 0.984 - 1.001 | 0.066 |  | 0.993 | 0.983 - 1.003 | 0.179 | 0.967 |
| % CX3CR1 | 1.002 | 0.993 - 1.010 | 0.728 |  | 1.000 | 0.990 - 1.009 | 0.939 | 0.992 |
|  |  |  |  |  |  |  |  |  |
| **% Transitional Monocytes (TM)** | 1.013 | 0.984 - 1.041 | 0.386 |  | 1.008 | 0.977 - 1.039 | 0.620 | 0.967 |
| % CCR2 | 0.996 | 0.981 - 1.011 | 0.574 |  | 0.994 | 0.977 - 1.012 | 0.539 | 0.967 |
| % CD11b | 0.964 | 0.841 - 1.105 | 0.598 |  | 0.941 | 0.806 - 1.099 | 0.444 | 0.967 |
| % CD40 | 0.998 | 0.980 - 1.016 | 0.795 |  | 1.014 | 0.993 - 1.034 | 0.185 | 0.967 |
| % CD86 | 1.008 | 0.984 - 1.033 | 0.502 |  | 1.025 | 0.994 - 1.058 | 0.120 | 0.967 |
| % CD163 | 0.899 | 0.694 - 1.165 | 0.421 |  | 0.973 | 0.731 - 1.296 | 0.852 | 0.967 |
| % CX3CR1 | 1.004 | 0.987 - 1.021 | 0.636 |  | 0.996 | 0.976 - 1.016 | 0.671 | 0.967 |
|  |  |  |  |  |  |  |  |  |
| **% CD14 Negative (HLA-DR+ CD14-)** | 1.010 | 0.976 - 1.046 | 0.565 |  | 0.997 | 0.960 - 1.035 | 0.876 | 0.967 |
| **% mDC** | 0.994 | 0.977 - 1.012 | 0.510 |  | 0.987 | 0.965 - 1.010 | 0.274 | 0.967 |
| % CCR2 | 0.997 | 0.982 - 1.011 | 0.655 |  | 0.993 | 0.974 - 1.013 | 0.493 | 0.967 |
| % CD11b | 1.002 | 0.986 - 1.020 | 0.777 |  | 1.002 | 0.977 - 1.028 | 0.871 | 0.967 |
| % CD40 | 0.995 | 0.980 - 1.011 | 0.562 |  | 1.002 | 0.984 - 1.021 | 0.808 | 0.967 |
| % CD86 | 0.984 | 0.962 - 1.006 | 0.144 |  | 0.977 | 0.950 - 1.005 | 0.111 | 0.967 |
| % CD163 | 0.972 | 0.553 - 1.710 | 0.922 |  | 0.874 | 0.503 - 1.519 | 0.633 | 0.967 |
| % CX3CR1 | 0.998 | 0.964 - 1.033 | 0.914 |  | 0.986 | 0.950 - 1.024 | 0.468 | 0.967 |
|  |  |  |  |  |  |  |  |  |
| **% CD123^dim^ CD11c^++^** | 1.014 | 0.983 - 1.046 | 0.391 |  | 1.019 | 0.982 - 1.059 | 0.321 | 0.967 |
| % CCR2 | 0.989 | 0.952 - 1.027 | 0.552 |  | 0.9653 | 0.917 - 1.011 | 0.130 | 0.967 |
| % CD11b | 1.003 | 0.988 - 1.019 | 0.656 |  | 1.000 | 0.981 - 1.020 | 0.973 | 0.992 |
| % CD40 | 0.994 | 0.978 - 1.010 | 0.465 |  | 1.004 | 0.982 - 1.026 | 0.734 | 0.967 |
| % CD86 | 0.996 | 0.966 - 1.026 | 0.780 |  | 0.992 | 0.953 - 1.032 | 0.685 | 0.967 |
| % CD163 | 1.054 | 0.946 - 1.175 | 0.340 |  | 1.018 | 0.904 - 1.146 | 0.773 | 0.967 |
| % CX3CR1 | 0.999 | 0.983 - 1.016 | 0.939 |  | 0.988 | 0.970 - 1.006 | 0.200 | 0.967 |
|  |  |  |  |  |  |  |  |  |
| **% pDC** | 1.045 | 0.883 - 1.237 | 0.609 |  | 1.080 | 0.870 - 1.339 | 0.486 | 0.967 |
| % CCR2 | 1.127 | 0.924 - 1.374 | 0.237 |  | 1.168 | 0.892 - 1.529 | 0.259 | 0.967 |
| % CD11b | 1.020 | 0.988 - 1.053 | 0.226 |  | 1.020 | 0.978 - 1.064 | 0.357 | 0.967 |
| % CD40 | 1.004 | 0.984 - 1.024 | 0.710 |  | 0.998 | 0.976 - 1.021 | 0.881 | 0.967 |
| % CD86 | 1.002 | 0.953 - 1.054 | 0.935 |  | 1.019 | 0.956 - 1.085 | 0.569 | 0.967 |
| % CD163 | 0.977 | 0.298 - 3.199 | 0.969 |  | 1.493 | 0.579 - 3.850 | 0.407 | 0.967 |
| % CX3CR1 | 0.718 | 0.344 - 1.500 | 0.378 |  | 0.401 | 0.153 - 1.048 | 0.062 | 0.967 |
|  |  |  |  |  |  |  |  |  |

**Supplementary Table 5.** Association between monocyte and dendritic cell phenotypes and their surface expression markers with cavitary disease among the total cohort.

|  | **Univariable** | | |  | **Multivariable** | | |  |
| --- | --- | --- | --- | --- | --- | --- | --- | --- |
|  | **OR** | **CI** | **p-value** |  | **OR** | **CI** | **p-value** | **q-value** |
| **% CD14 Positive (HLA-DR+ CD14+)** | 1.015 | 0.976 - 1.055 | 0.453 |  | 1.013 | 0.968 - 1.060 | 0.567 | 0.741 |
| **% Classical Monocytes (CM)** | 1.069 | 1.004 - 1.138 | **0.037** |  | 1.066 | 0.996 - 1.142 | 0.066 | 0.574 |
| % CCR2 | 1.017 | 0.999 - 1.036 | 0.066 |  | 1.009 | 0.989 - 1.029 | 0.400 | 0.660 |
| % CD11b | 1.059 | 0.846 - 1.327 | 0.615 |  | 1.066 | 0.828 - 1.372 | 0.619 | 0.753 |
| % CD40 | 0.973 | 0.930 - 1.019 | 0.246 |  | 0.985 | 0.936 - 1.037 | 0.565 | 0.741 |
| % CD86 | 1.053 | 1.000 - 1.109 | **0.049** |  | 1.054 | 0.994 - 1.117 | 0.079 | 0.574 |
| % CD163 | 1.009 | 0.986 - 1.033 | 0.456 |  | 1.012 | 0.989 - 1.038 | 0.385 | 0.660 |
| % CX3CR1 | 1.022 | 1.001 - 1.043 | **0.036** |  | 1.017 | 0.995 - 1.039 | 0.138 | 0.574 |
|  |  |  |  |  |  |  |  |  |
| **% Non-Classical Monocytes (NCM)** | 1.176 | 0.948 - 1.457 | 0.140 |  | 1.196 | 0.955 - 1.497 | 0.118 | 0.574 |
| % CCR2 | 0.988 | 0.921 - 1.061 | 0.745 |  | 0.964 | 0.891 - 1.043 | 0.368 | 0.660 |
| % CD11b | 1.019 | 0.988- 1.052 | 0.231 |  | 1.028 | 0.992 - 1.065 | 0.128 | 0.574 |
| % CD40 | 1.021 | 0.989 - 1.054 | 0.197 |  | 1.027 | 0.989 - 1.066 | 0.171 | 0.574 |
| % CD86 | 1.009 | 0.993 - 1.024 | 0.285 |  | 1.003 | 0.986 - 1.020 | 0.705 | 0.782 |
| % CD163 | 1.016 | 0.935 - 1.103 | 0.715 |  | 1.013 | 0.927 - 1.106 | 0.782 | 0.814 |
| % CX3CR1 | 1.039 | 1.006 - 1.073 | **0.022** |  | 1.031 | 0.993 - 1.070 | 0.107 | 0.574 |
|  |  |  |  |  |  |  |  |  |
| **% Intermediate Monocytes (IM)** | 4.612 | 1.011 - 21.041 | **0.048** |  | 4.693 | 0.912 - 24.156 | 0.064 | 0.574 |
| % CCR2 | 1.010 | 0.978 - 1.043 | 0.552 |  | 1.001 | 0.967 - 1.036 | 0.949 | 0.952 |
| % CD11b | 1.010 | 0.995 - 1.025 | 0.197 |  | 1.013 | 0.995 - 1.030 | 0.160 | 0.574 |
| % CD40 | 1.011 | 0.996 - 1.026 | 0.157 |  | 1.014 | 0.996 - 1.032 | 0.121 | 0.574 |
| % CD86 | 1.015 | 1.002 - 1.029 | **0.028** |  | 1.017 | 1.001 - 1.032 | **0.033** | 0.574 |
| % CD163 | 1.008 | 0.994 - 1.023 | 0.270 |  | 1.009 | 0.993 - 1.024 | 0.276 | 0.604 |
| % CX3CR1 | 1.011 | 0.998 - 1.026 | 0.106 |  | 1.009 | 0.993 - 1.024 | 0.272 | 0.604 |
|  |  |  |  |  |  |  |  |  |
| **% Transitional Monocytes (TM)** | 0.946 | 0.899 - 0.995 | **0.033** |  | 0.944 | 0.892 - 0.999 | **0.047** | 0.574 |
| % CCR2 | 1.025 | 0.999 - 1.052 | 0.059 |  | 1.014 | 0.986 - 1.043 | 0.332 | 0.650 |
| % CD11b | 1.078 | 0.893 - 1.302 | 0.434 |  | 1.058 | 0.855 - 1.309 | 0.607 | 0.753 |
| % CD40 | 0.979 | 0.952 - 1.006 | 0.130 |  | 0.987 | 0.957 - 1.018 | 0.411 | 0.660 |
| % CD86 | 1.009 | 0.969 - 1.051 | 0.667 |  | 1.008 | 0.964 - 1.053 | 0.729 | 0.791 |
| % CD163 | 1.253 | 0.817 - 1.922 | 0.301 |  | 1.353 | 0.840 - 2.180 | 0.213 | 0.574 |
| % CX3CR1 | 1.019 | 0.997 - 1.043 | 0.096 |  | 1.013 | 0.988 - 1.039 | 0.309 | 0.630 |
|  |  |  |  |  |  |  |  |  |
| **% CD14 Negative (HLA-DR+ CD14-)** | 0.946 | 0.894 - 1.000 | **0.048** |  | 0.945 | 0.890 - 1.005 | 0.071 | 0.574 |
| **% mDC** | 0.999 | 0.969 - 1.030 | 0.955 |  | 1.007 | 0.974 - 1.042 | 0.668 | 0.782 |
| % CCR2 | 1.026 | 0.998 - 1.056 | 0.073 |  | 1.015 | 0.984 - 1.048 | 0.344 | 0.650 |
| % CD11b | 1.025 | 0.994 - 1.057 | 0.118 |  | 1.024 | 0.989 - 1.059 | 0.179 | 0.574 |
| % CD40 | 0.976 | 0.950 - 1.002 | 0.070 |  | 0.983 | 0.954 - 1.012 | 0.251 | 0.604 |
| % CD86 | 0.986 | 0.952 - 1.022 | 0.446 |  | 0.983 | 0.942 - 1.026 | 0.427 | 0.660 |
| % CD163 | 0.845 | 0.371 - 1.923 | 0.688 |  | 0.800 | 0.332 - 1.930 | 0.620 | 0.753 |
| % CX3CR1 | 1.038 | 0.990 - 1.088 | 0.125 |  | 1.028 | 0.977 - 1.082 | 0.284 | 0.604 |
|  |  |  |  |  |  |  |  |  |
| **% CD123^dim^ CD11c^++^** | 1.054 | 0.995 - 1.118 | 0.074 |  | 1.048 | 0.984 - 1.115 | 0.145 | 0.574 |
| % CCR2 | 1.006 | 0.946 - 1.070 | 0.842 |  | 0.974 | 0.907 - 1.045 | 0.460 | 0.688 |
| % CD11b | 0.987 | 0.963 - 1.012 | 0.318 |  | 0.994 | 0.966 - 1.022 | 0.675 | 0.782 |
| % CD40 | 0.990 | 0.965 - 1.015 | 0.433 |  | 0.999 | 0.971 - 1.028 | 0.952 | 0.952 |
| % CD86 | 1.003 | 0.954 - 1.055 | 0.901 |  | 1.022 | 0.964 - 1.083 | 0.472 | 0.688 |
| % CD163 | 0.822 | 0.670 - 1.009 | 0.061 |  | 0.869 | 0.697 - 1.083 | 0.211 | 0.574 |
| % CX3CR1 | 1.019 | 0.995 - 1.044 | 0.126 |  | 1.012 | 0.984 - 1.040 | 0.417 | 0.660 |
|  |  |  |  |  |  |  |  |  |
| **% pDC** | 1.139 | 0.846 - 1.535 | 0.391 |  | 1.067 | 0.771 - 1.478 | 0.694 | 0.782 |
| % CCR2 | 1.136 | 0.837 - 1.541 | 0.414 |  | 1.126 | 0.790 - 1.604 | 0.513 | 0.727 |
| % CD11b | 0.990 | 0.928 - 1.056 | 0.754 |  | 1.021 | 0.951 - 1.095 | 0.565 | 0.741 |
| % CD40 | 1.008 | 0.975 - 1.042 | 0.639 |  | 1.027 | 0.985 - 1.070 | 0.214 | 0.574 |
| % CD86 | 1.084 | 0.955 - 1.230 | 0.211 |  | 1.092 | 0.956 - 1.247 | 0.194 | 0.574 |
| % CD163 | 0.421 | 0.087 - 2.046 | 0.284 |  | 0.353 | 0.057 - 2.167 | 0.261 | 0.604 |
| % CX3CR1 | 1.029 | 0.743 - 1.424 | 0.864 |  | 0.948 | 0.659 - 1.364 | 0.773 | 0.814 |

**Supplementary Table 6.** Association between monocyte and dendritic cell phenotypes and their surface expression markers with cavitary disease among the HIV positive individuals.

|  | **Univariable** | | |  | **Multivariable** | | |  |
| --- | --- | --- | --- | --- | --- | --- | --- | --- |
|  | **OR** | **CI** | **p-value** |  | **OR** | **CI** | **p-value** | **q-value** |
| **% CD14 Positive (HLA-DR+ CD14+)** | 1.019 | 0.976 - 1.065 | 0.395 |  | 1.016 | 0.959 – 1.077 | 0.588 | 0.780 |
| **% Classical Monocytes (CM)** | 1.051 | 0.977 - 1.131 | 0.182 |  | 1.054 | 0.971 – 1.144 | 0.211 | 0.644 |
| % CCR2 | 1.016 | 0.994 - 1.039 | 0.158 |  | 1.016 | 0.990 – 1.042 | 0.237 | 0.644 |
| % CD11b | 1.344 | 0.762 - 2.373 | 0.307 |  | 1.322 | 0.677 – 2.579 | 0.413 | 0.739 |
| % CD40 | 1.006 | 0.948 - 1.068 | 0.836 |  | 1.008 | 0.945 – 1.076 | 0.808 | 0.877 |
| % CD86 | 1.047 | 0.991 - 1.107 | 0.099 |  | 1.052 | 0.985 – 1.123 | 0.131 | 0.644 |
| % CD163 | 1.024 | 0.993 - 1.056 | 0.129 |  | 1.023 | 0.987 – 1.061 | 0.209 | 0.644 |
| % CX3CR1 | 1.015 | 0.991 - 1.041 | 0.225 |  | 1.015 | 0.987 – 1.043 | 0.302 | 0.644 |
|  |  |  |  |  |  |  |  |  |
| **% Non-Classical Monocytes (NCM)** | 1.134 | 0.911 - 1.413 | 0.261 |  | 1.181 | 0.921 – 1.514 | 0.190 | 0.644 |
| % CCR2 | 0.993 | 0.901 - 1.094 | 0.889 |  | 1.002 | 0.897 – 1.119 | 0.971 | 0.978 |
| % CD11b | 1.056 | 1.010 - 1.104 | **0.017** |  | 1.073 | 1.013 – 1.135 | **0.016** | 0.644 |
| % CD40 | 1.023 | 0.984 - 1.063 | 0.254 |  | 1.023 | 0.980 – 1.067 | 0.303 | 0.644 |
| % CD86 | 1.003 | 0.985 - 1.022 | 0.738 |  | 1.003 | 0.983 – 1.024 | 0.778 | 0.864 |
| % CD163 | 1.09 | 0.971 - 1.225 | 0.144 |  | 1.127 | 0.985 – 1.290 | 0.081 | 0.644 |
| % CX3CR1 | 1.024 | 0.982 - 1.068 | 0.265 |  | 1.019 | 0.971 – 1.070 | 0.434 | 0.739 |
|  |  |  |  |  |  |  |  |  |
| **% Intermediate Monocytes (IM)** | 3.506 | 0.729 - 16.868 | 0.118 |  | 5.389 | 0.810 – 35.829 | 0.081 | 0.644 |
| % CCR2 | 1.010 | 0.973 - 1.041 | 0.594 |  | 1.010 | 0.968 – 1.053 | 0.649 | 0.780 |
| % CD11b | 1.018 | 0.995 - 1.041 | 0.133 |  | 1.013 | 0.989 – 1.038 | 0.297 | 0.644 |
| % CD40 | 1.020 | 0.996 - 1.044 | 0.110 |  | 1.015 | 0.989 – 1.041 | 0.252 | 0.644 |
| % CD86 | 1.021 | 1.002 - 1.040 | **0.030** |  | 1.020 | 0.999 – 1.041 | 0.058 | 0.644 |
| % CD163 | 1.013 | 0.996 - 1.030 | 0.135 |  | 1.012 | 0.994 – 1.032 | 0.194 | 0.644 |
| % CX3CR1 | 1.011 | 0.993 - 1.029 | 0.226 |  | 1.011 | 0.992 – 1.030 | 0.270 | 0.644 |
|  |  |  |  |  |  |  |  |  |
| **% Transitional Monocytes (TM)** | 0.954 | 0.898 - 1.014 | 0.132 |  | 0.947 | 0.883 – 1.016 | 0.131 | 0.644 |
| % CCR2 | 1.015 | 0.985 - 1.046 | 0.330 |  | 1.015 | 0.977 – 1.054 | 0.444 | 0.739 |
| % CD11b | 1.165 | 0.895 - 1.516 | 0.257 |  | 1.202 | 0.857 – 1.684 | 0.286 | 0.644 |
| % CD40 | 0.999 | 0.965 - 1.034 | 0.947 |  | 1.003 | 0.963 – 1.043 | 0.900 | 0.937 |
| % CD86 | 1.014 | 0.967 - 1.062 | 0.572 |  | 1.029 | 0.970 – 1.092 | 0.336 | 0.661 |
| % CD163 | 1.400 | 0.834 - 2.349 | 0.203 |  | 1.490 | 0.841 – 2.640 | 0.171 | 0.644 |
| % CX3CR1 | 1.011 | 0.983 - 1.040 | 0.451 |  | 1.010 | 0.976 – 1.045 | 0.572 | 0.780 |
|  |  |  |  |  |  |  |  |  |
| **% CD14 Negative (HLA-DR+ CD14-)** | 0.942 | 0.880 - 1.008 | 0.086 |  | 0.942 | 0.873 – 1.015 | 0.117 | 0.644 |
| **mDC** | 1.010 | 0.974 - 1.048 | 0.578 |  | 1.027 | 0.979 – 1.077 | 0.280 | 0.644 |
| % CCR2 | 1.012 | 0.980 - 1.045 | 0.473 |  | 1.010 | 0.971 – 1.051 | 0.615 | 0.780 |
| % CD11b | 1.035 | 0.999 - 1.072 | 0.059 |  | 1.039 | 0.991 – 1.090 | 0.115 | 0.644 |
| % CD40 | 0.986 | 0.958 - 1.016 | 0.358 |  | 0.986 | 0.950 – 1.024 | 0.473 | 0.754 |
| % CD86 | 0.982 | 0.935 - 1.031 | 0.463 |  | 0.988 | 0.930 – 1.049 | 0.693 | 0.803 |
| % CD163 | 0.883 | 0.333 - 2.343 | 0.803 |  | 0.985 | 0.334 – 2.904 | 0.978 | 0.978 |
| % CX3CR1 | 1.029 | 0.967 - 1.095 | 0.365 |  | 1.041 | 0.969 – 1.118 | 0.270 | 0.644 |
|  |  |  |  |  |  |  |  |  |
| **% CD123^dim^ CD11c^++^** | 1.026 | 0.961 - 1.096 | 0.445 |  | 1.022 | 0.942 – 1.109 | 0.600 | 0.780 |
| % CCR2 | 0.970 | 0.896 - 1.050 | 0.456 |  | 0.977 | 0.894 – 1.068 | 0.614 | 0.780 |
| % CD11b | 1.008 | 0.977 - 1.040 | 0.625 |  | 1.010 | 0.972 – 1.049 | 0.617 | 0.780 |
| % CD40 | 1.009 | 0.977 - 1.043 | 0.569 |  | 1.008 | 0.972 – 1.047 | 0.658 | 0.780 |
| % CD86 | 1.020 | 0.957 - 1.086 | 0.548 |  | 1.039 | 0.961 – 1.124 | 0.337 | 0.661 |
| % CD163 | 0.871 | 0.704 - 1.078 | 0.204 |  | 0.874 | 0.684 – 1.118 | 0.285 | 0.644 |
| % CX3CR1 | 1.005 | 0.975 - 1.037 | 0.732 |  | 1.008 | 0.973 – 1.044 | 0.652 | 0.780 |
|  |  |  |  |  |  |  |  |  |
| **% pDC** | 1.142 | 0.798 - 1.634 | 0.469 |  | 1.061 | 0.701 – 1.607 | 0.779 | 0.864 |
| % CCR2 | 0.975 | 0.646 - 1.473 | 0.905 |  | 0.879 | 0.531 – 1.455 | 0.616 | 0.780 |
| % CD11b | 1.026 | 0.953 - 1.105 | 0.492 |  | 1.034 | 0.948 – 1.127 | 0.449 | 0.739 |
| % CD40 | 1.019 | 0.976 - 1.063 | 0.397 |  | 1.013 | 0.964 – 1.065 | 0.604 | 0.780 |
| % CD86 | 1.170 | 0.960 - 1.427 | 0.119 |  | 1.173 | 0.919 – 1.498 | 0.201 | 0.644 |
| % CD163 | 0.374 | 0.048 - 2.931 | 0.349 |  | 0.325 | 0.018 – 5.836 | 0.445 | 0.739 |
| % CX3CR1 | 0.925 | 0.233 - 3.680 | 0.912 |  | 1.125 | 0.244 – 5.178 | 0.880 | 0.935 |
